# Biology of Bony Fish Macrophages

**DOI:** 10.3390/biology4040881

**Published:** 2015-11-30

**Authors:** Jordan W. Hodgkinson, Leon Grayfer, Miodrag Belosevic

**Affiliations:** 1 Department of Biological Sciences, University of Alberta, Edmonton, AB T6G 2E9, Canada; E-Mail: mbelosev@ualberta.ca; 2 Department of Biological Sciences, George Washington University, Washington, DC 20052, USA; E-Mail: leon_grayfer@email.gwu.edu

**Keywords:** teleost, macrophages, antimicrobial, cytokine, respiratory burst, nitric oxide, nutrient depravation

## Abstract

Macrophages are found across all vertebrate species, reside in virtually all animal tissues, and play critical roles in host protection and homeostasis. Various mechanisms determine and regulate the highly plastic functional phenotypes of macrophages, including antimicrobial host defenses (pro-inflammatory, M1-type), and resolution and repair functions (anti-inflammatory/regulatory, M2-type). The study of inflammatory macrophages in immune defense of teleosts has garnered much attention, and antimicrobial mechanisms of these cells have been extensively studied in various fish models. Intriguingly, both similarities and differences have been documented for the regulation of lower vertebrate macrophage antimicrobial defenses, as compared to what has been described in mammals. Advances in our understanding of the teleost macrophage M2 phenotypes likewise suggest functional conservation through similar and distinct regulatory strategies, compared to their mammalian counterparts. In this review, we discuss the current understanding of the molecular mechanisms governing teleost macrophage functional heterogeneity, including monopoetic development, classical macrophage inflammatory and antimicrobial responses as well as alternative macrophage polarization towards tissues repair and resolution of inflammation.

## 1. Introduction

Macrophage lineage cells present a remarkably versatile array of functional specializations across vertebrates. As resident cells in virtually all tissues, macrophages aid in maintaining homeostatic environments, and upon infection, are typically one of the first cell types to encounter intruding pathogens, where they orchestrate appropriate immune responses. Our understanding of macrophage biology has greatly expanded since the first description of starfish larvae phagocytes in 1882 by Élie Metchnikoff, who was later awarded the Nobel Prize for his contribution to cellular immunity in 1908 [1]. Since then, the macrophage has emerged as an essential cell type in all vertebrates, endowed with a panoply of functional capabilities.

Much of our understanding of macrophage biology comes from research in mammalian models, where distinct macrophage subsets of have been characterized, including classically activated cells by interferons (IFN) and tumor necrosis factor alpha (TNFα) (M1); alternatively activated cells by IL-4 and IL-13 (M2a); macrophages activated by immune complexes or apoptotic cells (M2b); and regulatory macrophages, deactivated by IL-10, TGF-β, or glucocorticoids (M2c), which culminate in the various effector subtypes, broadly described as having a “kill” or “heal” response (reviewed in [2]). Recent transcriptomic and proteomic analyses of macrophage populations derived by these distinct stimuli have underlined the vast complexities of these mechanisms at the molecular level, controlling the various physiological responses of macrophages.

In teleosts, the best characterized macrophage phenotype is that comparable to the M1 activation state, which serves a critical role in host protection. These cells may rapidly kill pathogens by engulfment and production of toxic reactive intermediates [3], phagolysosomal acidification [4], and restriction of nutrient availability [5]. Furthermore, M1 macrophages are robust factories of cytokines, chemokines, and lipid mediators, which act to potentiate and fine-tune the inflammatory and adaptive immune responses. More recently, efforts to characterize alternative activation states of teleost macrophages are focusing on the biology of fish IL4 and IL13 homologues (IL4/13A and IL4/13B) and arginase activity, implying a conserved M2a phenotype [6,7]. Similar deactivating roles of glucocorticoids (GC), immune complexes, IL-10, and TGF-β have been demonstrated in teleosts, suggesting conserved functions of these ligands in deactivating or aiding in the tissue repair [8,9,10]. Characterization of the regulatory mechanisms responsible for shaping macrophage polarity is a unique challenge in teleosts, as whole-genome duplication and gene-specific duplication events, combined with disparate evolutionary pressures, have endowed distinct teleosts with multiple gene copies, the product of some of which may have adopted respectively divergent roles [11].

This review discusses bony fish macrophage development, polarization, and functional responses, thus comprehensively coalescing the current understanding of teleost macrophage biology.

## 2. Macrophage Development

### 2.1. Teleost Embryonic Monopoiesis

Akin to most other vertebrates studied to date, teleost fish blood cell development occurs within primitive and definitive waves of hematopoiesis [12,13,14]. Over the last few decades, the zebrafish has emerged as a model of choice to study blood cell formation, owing to its optical transparency and relative ease of genetic manipulation. During primitive hematopoiesis (12–24 h post-fertilization), embryonic mesoderm is committed to monopotent hematopoietic precursors that give rise to macrophages, the first functional blood cell type, in the rostral blood island of the zebrafish embryo [12,14,15]. Following primitive monopoiesis, a transient definitive hematopoietic wave is initiated in the posterior blood island, giving rise to the first multilineage progenitor cells, erythromyeloid progenitors (EMPs), which develop into both erythroid and myeloid cells. Definitive hematopoiesis, defined by the emergence of multipotent hematopoietic stem cells (HSCs), subsequently originates in the aorta-gonad-mesonephros (AGM), and HSCs that precede monopoeisis are seeded into caudal and renal tissue, serving as the main sources of myeloid cells roughly 3–4 days post-fertilization (dpi) [16]. The existence of renal marrow-derived HSCs has been demonstrated in both zebrafish and gibuna carp, where transplantation of renal HSCs from healthy donors to irradiated recipients reconstituted HSC pools of the stem cell-depleted fish [17,18]. The complex and intricate processes described above are more comprehensively reviewed in [13,16,19,20].

### 2.2. Teleost Adult Monopoiesis

For nearly a half a century, the widely accepted mononuclear phagocyte system theory proposed that tissue macrophages are derived from circulating blood monocytes in vertebrates [21]. Although it is well established that monocytes give rise to macrophages during inflammatory conditions in both mammals and fish, recent evidence by fate-mapping blood cell lineages suggests a more limited contribution of circulating monocytes to mature tissue macrophage replenishment. Instead, tissue macrophages are “seeded” during primary hematopoiesis and self-maintain resident populations, as demonstrated in mammals [22,23,24]. This developmental feature has also been observed in fish lacking functional *c-myb*, resulting in tissue macrophage development in the absence of definitive hematopoiesis [25].

### 2.3. Roles of Colony-Stimulating Factor-1 in Teleost Monopoieis

Across all vertebrate species examined to date, the survival, proliferation, differentiation, and functionality of most cells of the macrophage lineage are governed by the cytokine colony-stimulating factor-1 (CSF-1 = macrophage-colony-stimulating factor, M-CSF) [26,27,28,29] through binding to its cognate receptor (CSF-1R) [30]. In turn, the CSF-1R is expressed almost exclusively on committed myeloid precursors and derivative macrophage populations [31,32].

From a phylogenetic perspective, it is notable that birds and mammals possess a single, alternatively spliced CSF-1 gene that produces both membrane-bound and secretory forms of this cytokine [33,34]. Intriguingly, several teleost fish species have been documented to possess two distinct CSF-1 genes, which at present are not thought to be alternatively spliced [29]. Akin to the mammalian CSF-1, the fish counterpart is also a proficient macrophage growth and differentiation factor [35,36]. It is, however, notable that unlike the M2-potentiating roles of the mammalian CSF-1 [37], the functional work performed on one of the two cyprinid CSF-1 molecules suggests that the teleost counterpart cytokine differentiates M1-like kidney macrophages, exhibiting upregulated pro-inflammatory components [36]. This is further supported by the recent findings that a novel soluble goldfish CSF-1R serves to ablate fish pro-inflammatory responses by reducing available soluble CSF-1 [38]. It will be interesting to determine whether the respective fish CSF-1 proteins confer the biological roles of mammalian CSF-1 splice variants, or if these moieties have adopted novel roles in respect to the M1/M2 macrophage paradigm.

The mammalian macrophage lineage cell CSF-1R gene expression increases progressively with cell development, with committed myeloid precursors possessing the lowest transcript levels of this receptor, monocytes and immature macrophages expressing significantly greater levels of the CSF-1R, and mature macrophages exhibiting still greater levels of the CSF-1R transcript [39]. Moreover, cell-signaling through the CSF-1R not only governs macrophage proliferation, differentiation, and survival, but also macrophage antimicrobial responses [40,41,42,43]. It is intriguing that teleosts possess multiple CSF-1 genes and at least some fish species, such as Fugu, also express two distinct CSF-1R genes [44]. While it is presently unknown why fish have adopted unique CSF-1/CSF-1R orthologues, it is compelling to consider that these distinct ligand-receptor combinations may facilitate unique teleost monopoietic consequences unique from those of mammals. Such deviations have already been documented for other teleost cytokine-receptor systems, such as the type II IFNs [45,46,47,48], and render it more likely that the multiple fish CSF-1s and CSF-1Rs may also possess complex interactions, distinct from those generally seen across endothermic species.

## 3. Classical Macrophage Activation

### 3.1. Molecular Mechanisms of Classical Activation

#### 3.1.1. Type II Interferons

Macrophage activation was first coined by Mackaness in the 1960s after describing antimicrobial activity of mouse macrophages to secondary infection with intracellular bacteria [49]. Later, this phenomenon of cellular resistance resulting in the antimicrobial state of macrophages was referred to as “classical activation”, or M1, corresponding to the general effector phenotype of these phagocytes during cell-mediated (Th1) immune responses [50]. M1 polarization predominantly refers to macrophages activated by the Th1-derived cytokines, and chiefly IFNγ, which is provided transiently early on by NK cells and more prominently during the adaptive immune response by Th1 helper cells [51,52]. In addition to IFNγ, co-stimulation by TNFα is necessary to fully induce M1 macrophage microbicidal activity [53]. It is important to note that M1 polarization can be driven by microbial stimuli alone through pattern recognition receptor (PRR) activation and in the absence of exogenous cytokine stimulation. Notably, MyD88-dependant toll-like receptor (TLR) engagement leads to transcription of TNFα, in addition to IFNβ expression independent of MyD88 TLR signaling [54], enabling macrophage autocrine stimulation. Indeed, fish species possess extensive arrays of pattern recognition receptors, both putative mammalian orthologues and fish-specific family members [55], and have been shown to activate with microbial stimulus in the absence of exogenous cytokine addition.

In fish, type II interferons have been well characterized [56,57]. IFNγ has been sequenced in fugu [58], rainbow trout [59], zebrafish [60], Atlantic salmon [52], catfish [61], common carp [62], goldfish [63], Atlantic cod [64], and flounder [65]. Recombinant IFNγ has been shown to elicit STAT1 signaling in head kidney leukocytes of Atlantic salmon [66] and culminates in M1-like macrophages of goldfish, grass carp, and common carp, indicating a possible functional conservation to the mammalian counterpart [46,67,68].

Interestingly, certain fish species possess at least two distinct type II IFNs, termed IFNγ and IFNγ-related (IFNγrel). Recombinant IFNγrel has been shown to activate macrophage reactive intermediate production [63] in goldfish, and recombinant IFNγrel molecules enhance antiviral activity in gibuna carp [47]. Furthermore, partially overlapping roles of IFNγ and IFNγrel in knockout zebrafish [69] indicate a redundancy of IFN function for possible M1 polarization in fish. While these type II IFNs have been identified in several orders of teleosts, the majority of the functional studies of fish IFNγ and IFNγrel have been performed in cyprinids. In this respect, the injection of zebrafish embryos with IFNγ or IFNγrel mRNAs individually elicits comparable immune gene expression profiles, and combined injections further enhance certain gene expression profiles, possibly owing to the non-overlapping roles of the respective cytokines [69]. Interestingly, when either the zebrafish IFNγ or IFNγrel is individually knocked down, zebrafish embryo survival following *Escherichia coli* challenge is uncompromised, whereas knock-down of both IFNγs results in significant increases in infection-induced mortalities [69]. Notably, IFNγ or IFNγrel morpholino knock-downs decrease embryo survival following *Yersinia ruckeri* infections, while knock-down of both IFNs further compounds embryo mortalities. Presumably, IFNγ and IFNγrel induce both overlapping and distinct antimicrobial mechanisms.

We have documented that the recombinant forms of the goldfish (rg) IFNγ and rgIFNγrel have very different effects on kidney macrophage functions [63]. While rgIFNγ evokes long-lasting reactive oxygen intermediate (ROI) priming, rgIFNγrel-induced ROI priming is short-lived and is followed by unresponsiveness of the stimulated phagocytes to ROI induction by other M1 cytokines (rgIFNγ or rgTNFα2). Moreover, rgIFNγrel appears to be a much more potent inducer of macrophage phagocytosis and nitric oxide (NO) production than rgIFNγ, and the two cytokines induce distinct expression levels of key macrophage immune genes [46,70,71]. Finally, it would appear that the downstream signaling mechanisms employed by these type II IFNs are also distinct, with IFNγ more closely resembling the mammalian IFNγ signaling.

Further confirmation of this functional dichotomy between the fish type II IFNs is warranted using *in vivo* and other *in vitro* fish models. Notably, the zebrafish IFNγrel has recently been shown to elicit more robust pro-inflammatory gene expression than IFNγ in larvae microinjected with respective IFN expression constructs [72]. Interestingly, these zebrafish IFNγrel-mediated effects were dependent on the myeloid transcription factor SP1, underlying the specificity of this cytokine for macrophage lineage cells.

In comparison to other vertebrates that possess a single IFNγ receptor 1 (IFNGR1), it is particularly notable that certain teleosts possess two distinct IFN gamma-receptor binding chains (IFNGR1-1 and IFNGR1-2) that confer distinct but poorly understood interactions with the IFNγ and IFNγrel molecules of these respective species [45,48,63]. Together, it would appear that fish have adopted very unique strategies with respect to the principal M1 macrophage activating cytokine system. It will be interesting to further examine how these respective type II IFNs participate in teleost macrophage functional polarization.

#### 3.1.2. Tumor Necrosis Factor-Alpha

Akin to many fish cytokines, multiple isoforms of the key inflammatory cytokine, tumor necrosis factor alpha (TNFα), have been documented across numerous fish species [73,74,75,76,77,78,79,80,81,82,83,84]. In goldfish and carp, TNFα1 and TNFα2 have been implicated as having pro-inflammatory roles, and enhancing pro-inflammatory gene expression, as well as phagocytic, reactive oxygen, and nitrogen intermediate production, akin to the pro-M1 roles of the mammalian TNFα [59,74]. Recently, transgenic zebrafish bearing a fluorescent TNFα reporter were shown to recruit a subset of macrophages during both aseptic wounding and wounding with *E. coli* inoculation, where TNFα expression occurred in the presence of bacteria, thus underlining the microbial activation and M1 polarization of macrophages [85]. TNFα3 expression is increased following stimulation of HK macrophages with PAMPs, and rTNFα3 increases production of a number of pro-inflammatory cytokines, although the functional relevance of this gene has yet to be fully elucidated [84].

#### 3.1.3. Granulocyte-Macrophage Colony-Stimulating Factor

Granulocyte macrophage colony-stimulating factor (GM-CSF) has been added to the list of M1 stimuli in mammals [86]. GM-CSF aids in the survival, proliferation, and differentiation during myelopoiesis through receptor chains CSF2Rα and CSF2Rβ [87]. GM-CSF has not yet been confirmed in teleosts, although recent discovery of a GM-CSF-like molecule in the elephant shark may infer possible existence in bony fish [88]. Moreover, cDNA and gene sequences of numerous teleost fish CSF2Rβ are available on GenBank, further substantiating the possibility of a teleost GM-CSF, although it should be noted that in mammals this receptor chain is shared by GM-CSF, IL-3, and IL-5 [89], inferring a possibility of disparate ligand interactions in lower vertebrates.

### 3.2. M1 Macrophage Cytokine Profiles

Stimulation and polarization of macrophages to an M1 subtype results in the production of large amounts of TNF, IL-1β, IL-6, IL-12 IL-15, and IL-23, for further activation of proximal cell types, aiding chemotaxis of inflammatory leukocytes (*i.e.*, neutrophils and monocytes), and influencing Th1 polarization, which in turn amplifies classical activation [90]. This main M1 cytokine repertoire is present in fish, and has been demonstrated to up-regulate following microbial challenge, as well as following stimulation with M1-inducing stimuli, akin to that of mammals [10,67,74,91]. The detailed repertoire of cytokine networks, including Th1 cytokine networks of fish, has been comprehensively reviewed [57].

## 4. Antimicrobial Roles of Teleost M1 Macrophages

### 4.1. Respiratory Burst Response

The destruction of internalized microorganisms is key to the innate immune response. It is well established that fish phagocytes, including macrophages, generate reactive oxygen intermediates as an antimicrobial defense, similar to mammalian phagocytes. During the macrophage respiratory burst, an assembly of multicomponent enzyme nicotinamide adenine dinucleotide phosphate (NADPH) subunits at the plasma membrane results in the transfer of electrons from NADPH to molecular oxygen, resulting in the superoxide anion [92]. Upon formation, superoxide rapidly converts to reactive oxygen species (ROS) hydrogen peroxide (H_2_O_2_), hydroxyl radical (OH·), and hyperchlorous acid (HOCl), which efficiently kill microorganisms [93,94] (El-Benna *et al.*, 2008). The NADPH oxidase complex is made up of six individual subunits that are segregated in resting cells, including the cytosolic components p40phox (phagosome oxidase), p47phox, p67phox, and a guanosine triphosphatase (GTPase) Rac 1 or Rac 2 migrates to membrane-associated subunits gp91phox (also known as Nox2) and p22phox [95,96,97,98,99,100], all of which have been cloned in several fish species [101]. Despite low sequence homology between fish and mammals, the functional sites of NADPH-oxidase are highly conserved [102,103]. ROI production by teleost macrophages has been observed following stimulation with pathogen-associated molecular patterns (PAMPs) [4,104,105,106,107], fish pathogens [108,109,110], and recombinant cytokines such as TNFα [74,80,111], IFNγ [46,59,67] and CSF-1 (MCSF). Notably and as described above, this is in contrast to the mammalian CSF-1, which is considered to be an M2 stimulus, opposite to GM-CSF [86]. The importance of respiratory burst to host protection is underlined by the various pathogens that can effectively ablate or withstand toxic oxygen intermediates. For example, *Edwardsiella tarda*, a natural pathogen of various economically important fish and the etiological agent of edwardsiellosis, has several strategies to combat ROI production of teleost macrophages, reviewed by [5].

### 4.2. Nitric Oxide Response

Akin to superoxide production, the inducible nitric oxide (NO) system of teleost macrophages is well conserved compared to those described in mammals. Classically activated macrophages are distinguished by the expression of inducible nitric oxide synthase (iNOS/NOS2) that catalyzes the conversion of l-arginine to l-citruline, resulting in production of NO, a potent antimicrobial compound [112,113]. Simultaneous production of superoxide and NO intermediates can also form peroxynitrite (ONOO^−^), which additionally serves potent antiparasitic/antimicrobial functions [114,115]. Indeed, iNOS is a prototypical marker of M1 activation in macrophages that is readily up-regulated in response to IFNγ, TNFα, and microbial compounds (e.g., LPS) [51].

The fish iNOS has been characterized with marked similarity to the mammalian enzyme counterpart, possessing the putative binding sites for heme, tetrahydrobiopterin, calmodulin, flavine mononucleotide, flavine adenine dinucleotide, and NADPH, suggesting cofactor conservation and function [81]. First identified as a partial sequence in goldfish [116], iNOS has been identified and characterized in carp [81], rainbow trout [117], zebrafish [118], and turbot [119]. The gene expression of fish macrophage iNOS coincides with cellular NO production, and has been demonstrated to increase following exposure to PAMPS or a microbial stimulus [6,81,120] and pro-inflammatory cytokine stimulus [35,46,67,74,80,83,121], as well as cleaved transferrin products [122]. The importance of iNOS in host protection has been implicated in the control of many varieties of fish pathogens, including viral hemorrhagic septicemia virus (VHSV), *Aeromonas salmonocida*, *Renibacterium salmoninarum*, *Yersinia ruckeri* [123,124,125], and *Mycobacterium marinum* [91,126,127,128].

### 4.3. Phagolysosome Fusion

Following pathogen uptake, the phagosome undergoes various maturation steps that are an important process to destroy the internalized microbe, which culminates in phagolysosome formation. Both M1- and M2-type macrophages rapidly form phagolysosomes in order to degrade internalized material. Interestingly, recent reports of M1-stimulated macrophages show relatively neutral pH following the internalization of zymosan, in contrast with M2-driven macrophages that rapidly acidify their phagosomes [129]. This is presumably due to the proton consumption during superoxide production, a feature that may also aid in preserving antigen for presentation by decreasing the degradative capacity, as is seen in mammalian dendritic cells [130]. Not surprisingly, fish monocytes and macrophages demonstrate the ability to undergo phagolysosomal fusion [4]. Additional work is necessary to elucidate the importance of phagosome maturation of fish macrophages in host immune defenses with regard to macrophage polarization.

### 4.4. Nutrient Deprivation

#### 4.4.1. Solute Carrier 11 Member 1

Solute carrier 11 member 1, Slc11a1 (formally known as natural resistance-associated macrophage protein 1, NRAMP1), is a divalent metal ion transporter, present in the late endosomes and lysosomes of professional phagocytes [131]. Transcriptional regulation of Slc11a1 is induced by hypoxia-inducible factor alpha (HIFα), predominantly active in M1 macrophages in response to microbial or pro-inflammatory cytokine stimuli [132,133]. Mutations in the Slc11a1 allele confer susceptibility to infection with intracellular pathogens, including *Salmonella*, *Leishmania*, *and Mycobacteria* [134]. Although there is a clear role of NRAMP1 in innate immunity, the precise antimicrobial mechanisms conferred by this ion channel remain elusive. Evidence of movement of Fe^2+^ into the phagosome in RAW264.7, a NRAMP1-deficient cell line, shows iron transport into phagosomes, which may aid in catalyzing ROS and limiting bacterial growth [135]. Contrary to these findings, reports of metal movement out of the phagosome, which is proposed to limit growth by restricting nutrients, has also been observed [136,137], leading to the proposal of Slc11a1 as a pH-dependent bidirectional transporter [138].

NRAMP1 has been cloned in a number of fish species, including carp [139], fugu [140], channel catfish [141], rainbow trout [142], turbot [143], sea bream [144], Japanese flounder [145], and striped bass [144]. Although orthologs of Slc11 have been identified in several fish species, the phylogeny, sequence identities, and expression patterns of these teleost NRAMP orthologs do not clearly indicate whether these primordial counterparts are specifically orthologous to Slc11a1 or Slc11a2. Despite this, several studies have indicated that the fish NRAMP counterparts are intimately involved in innate immune responses against pathogens.

Teleost fish appear to possess multiple Slc11 orthologs per genome including two fugu Slc11a genes [140] and three catfish Slc11a orthologs [141]. Moreover, while the phylogenetic relationships between the mammalian and fish Slc11 genes remain to be defined, the teleost NRAMP counterparts are indeed directly involved in antimicrobial responses against fish pathogens such as *V. anguillarum* [144], *E. ictaluri* [146], and *M. marinum* [91,147].

#### 4.4.2. Ferroportin

The first identification of ferroportin (also known as metal-transporting protein-1 or iron-regulated transporter-1) was in zebrafish [148] and mice [149]. Activation with IFNγ, LPS, or intracellular pathogens has been shown to enhance ferroportin expression, which reduces intracellular iron, thereby depriving the pathogen of a necessary enzymatic cofactor [150,151,152], indicating an important role of this protein in M1-activated macrophages. The overexpression of ferroportin has been shown to disrupt the intracellular growth of *Mycobacteria* and *Salmonella*, and ferroportin-deficient mice are more susceptible to intracellular pathogens [153]. The control of ferroportin is predominantly mediated by hepcidin (up-regulated in mammals by IL-6), which binds to ferroportin and promotes internalization and degradation in lysosomes, diminishing the release of iron from macrophages [154].

Ferroportin has been identified in zebrafish [148] and turbot [155], showing correlated expression levels during *V. anguillarum* infections. Interestingly, the zebrafish mutant *weissherbst* is a hyperchromatic blood mutant with a mutation in functional ferroportin [148], which may be valuable in further elucidating the effects of ferroportin in teleosts.

#### 4.4.3. Indoleamine 2,3-Dioxygenase

Indoleamine 2,3-dioxygenase (IDO), or tryptophan 2,3 dioxygenase (IDO), is a macrophage enzyme that catalyzes the degradation of tryptophan, and is up-regulated in IFNγ-stimulated macrophages [156]. M1 macrophage IDO expression and activation of this immunoregulatory and antimicrobial mechanism is intimately linked to cell stimulation by IFNγ [157]. IDO tryptophan degradation results in the production of a panel of immunoregulatory metabolites, collectively referred to as kynurenins [158]. In turn, the IDO-derived kynurenins promote immunotolerance and suppress excessive proliferation of activated cytotoxic leukocytes. Presently, the mechanisms governing M1 macrophage IDO are poorly defined; however, it is thought that the IFNγ-mediated activation of IDO serves as a nutrient deprivation mechanism by reducing and preventing tryptophan supply to intracellular or locally detected pathogens [159,160,161,162].

Bony fish IDO orthologs appear to possess very low tryptophan-degradative enzymatic efficiencies compared to the mammalian IDOs [163], suggesting the presence of alternative fish IDO substrates, and were aptly renamed as proto-IDOs. Interestingly, marsupials possess both IDO and proto-IDO, in tandem on a single chromosome, whereas only proto-IDOs are found in fish, amphibians, and chickens, implying that the mammalian IDO arose from a gene duplication event of the proto-IDO.

**Figure 1:**
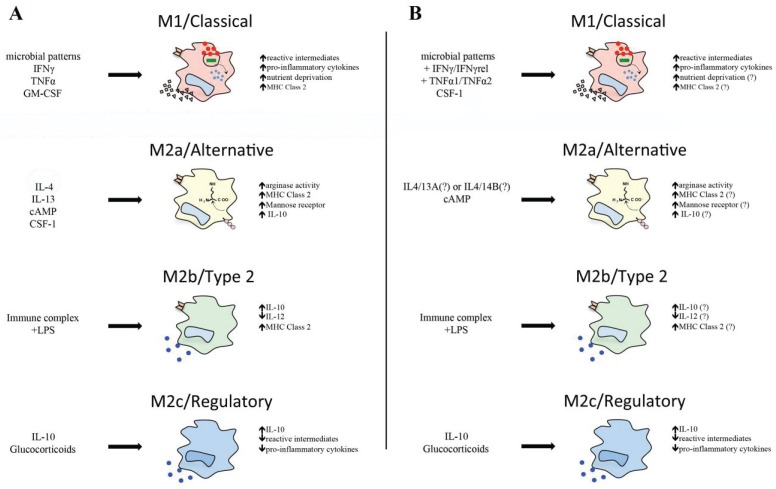
(**A**) Activation states of mammalian macrophages. Stimulation with microbial patterns in addition to type II interferons, TNFα or GM-CSF, leads to increased antimicrobial responses of macrophages, pro-inflammatory cytokine production, and nutrient deprivation in accordance with an M1 activation profile. Alternative activation of macrophages by cAMP IL4 and/or 13 ligands results in increased arginase expression and activity. Stimulation with immune complexes and lipopolysaccharides (LPS) generates a unique IL-10^high^ IL-12^low^ M2b phenotype. IL-10 or glucocorticoid-activated macrophages drive regulatory function, inhibiting pro-inflammatory cytokine production, antimicrobial activity, and driving IL-10 expression; (**B**) Activation states of teleost macrophages. Stimulation with microbial patterns, type II interferons, TNFα isoforms, or CSF-1 leads to increased antimicrobial responses similar to an M1 phenotype. Alternative activation of teleost macrophages can be achieved by cAMP stimulation. Immune complex, IL-10, and glucocorticoids can deactivate teleost macrophages. Question marks refer to molecules that are present in teleosts, but have yet to be linked to alterations in macrophage plasticity.

Notably, goldfish macrophages infected with *M. marinum* significantly up-regulate their proto-IDO gene expression, where live *M. marinum* induces substantially greater proto-IDO transcript levels than the heat-killed bacteria [91]. This suggests that the fish proto-IDO expression is possibly advantageous to *M. marinum* survival in macrophages, and may indicate that this fish proto-IDO serves to dampen immune cell bystander responses, akin to its mammalian counterpart. A summary of mammalian and teleost M1 macrophage function is depicted in Figure 1.

## 5. Alternative Teleost Macrophage Activation

### 5.1. Interleukin-4/13

M2 macrophages are generally considered as being antagonists to M1 macrophages, having “anti-inflammatory” or “pro-healing” functions. In mammals, the best characterized M2 stimuli are IL-4 and IL-13 (sometimes called M2a), produced by Th2 cells, eosinophils, basophils, NKT cells, and macrophages themselves [164]. The recognition of IL-4 by receptor pairs IL-4Rα combined with either IL4γc or IL-13α1 chains results in signaling through JAK1 and JAK3, leading to STAT6 activation and nuclear translocation. IL-13 can also bind the IL13Rα2 chain, which has not been fully characterized [165]. Mouse and human macrophage proteins up-regulated in response to these M2 stimuli include: transglutaminase 2 (TGM2), macrophage mannose receptor (MMR1/MRC1), cholesterol hydroxylase CH25H, prostaglandin-endopreoxide synthase (PTGS1), transcription factors IRF4 and Kruppel-like factor 4 (KLF4), and signaling molecules with cytokine-inducible SRC homology 2 (SH2) containing protein (CISH) and suppressor of cytokine signaling 1 (SOCS1), all of which are present in teleost fish, although have yet to be described as M2 markers [105,166,167,168,169,170].

To date, at least two genes have been identified in fish that share homology with both the mammalian IL-4 and IL-13 cytokines (IL-4/13A and IL-4/13B) [171], although genome/gene duplication events have resulted in variable numbers of these gene copies in different fish species [172]. Two paralogs of IL-4Rα, IL-13Rα1, and IL-13Rα2 have been identified in rainbow trout and zebrafish [173,174]. Although there is no information on IL-4/IL13 alternative activation of fish macrophages, recombinant IL-4/13A has been shown to bind IL-13Rα [174], and was shown to support B and T cell expansion, indicative of a conserved role for this cytokine in fish Th2 adaptive immunity [175,176]. It will be interesting to learn whether the IL-4/13 cytokines serve to establish M2 macrophage phenotypes in lower vertebrates, including fish.

### 5.2. Arginase

A major bifurcation of M1 and M2 function involves the metabolism of l-arginine. Whereas iNOS of M1 macrophages converts l-arginine to l-citruline and NO, arginase, a putative marker of M2 macrophages, converts l-arginine to l-ornithine and urea [177,178,179,180]. The repair phenotype of M2 macrophages is predicated by the production of l-ornithine, which is a precursor for polyamines and proline components of collagen that are important for tissue repair [181]. Interestingly, byproducts of either iNOS or arginase pathways inhibit the reciprocal enzymes, thus stabilizing the M1 or M2 macrophage polarization states, respectively [182].

Mammals possess two arginase isoforms including arginase-1, located in the cytosol and induced by IL-4 and IL-13 [183], and arginase-2, associated with the mitochondria and up-regulated by IL-10 and LPS [184]. In fish, arginase-1 and arginase-2 were first identified in rainbow trout, phylogenetically clustering with the respective mammalian orthologues [185]. Arginase activity has been demonstrated in cyclic adenosine monophosphate (cAMP)-stimulated murine macrophages which increased intracellularly during the IL-13 signal transduction [186]. Similarly, common carp macrophages stimulated with cAMP show increased arginase activity and specific inhibition of urea production with *N*^G^-hydroxy-l-arginine, suggesting an evolutionary conservation of polarized macrophages in lower vertebrates [6]. Indeed, work using the carp kidney macrophage model has established that, like mammalian cells, classically polarized fish macrophages are marked by high iNOS gene expression, whereas alternatively polarized macrophages are distinguishable by highly up-regulated arginase gene expression [6,10,120]. Intriguingly, in contrast to the mammalian M2 macrophage arginase-1 expression, carp alternative macrophage activation coincides with increased arginase-2 transcript levels [6]. For excellent comprehensive reviews of alternative fish macrophage polarization and the fish arginase responses, refer to recent reviews [187,188].

### 5.3. Immune Complex and Lipopolysaccharides

Repeated stimulation of M1-like macrophages by inflammatory stimuli has been documented to result in the adoption of M2-like macrophage phenotypes and unresponsiveness to subsequent inflammatory cues. For example, mammalian M2b or type-2 macrophages are generated in response to immune complexes (IC) and Gram-negative bacteria lipopolysaccharides (LPS), resulting in lower IL-12 and higher IL-10 production [164]. This is thought to be an essential link in dampening early M1 activity during inflammation, and thus promoting tissue remodeling and regeneration [189]. Carp blood parasite infection studies additionally demonstrated this phenomenon, showing that *Trypanoplasma borreli* immune complex formation generated distinctly activated carp macrophage subsets, promoting the resolution of inflammation and parasite clearance [10].

### 5.4. Glucocorticoids and Interleukin-10

Deactivation of macrophages by glucocorticoids (GCs) and IL-10 has also been described to culminate in the unique M2c macrophage activation state, otherwise referred to as “regulatory macrophages”. The diffusion of GCs across the plasma membrane leads to interaction with the glucocorticoid receptor (GCR), which results in nuclear translocation and direct transcriptional up-regulation of some immune genes and down-regulation of others, resulting in macrophage phenotypes and transcriptional profiles distinct from those observed following IL-4 macrophage stimulation [190,191]. For example, GC stimulation of macrophages antagonizes classically activated macrophage functions, including down-regulation of inflammatory cytokines and dampening of reactive intermediate production. Cortisol has been shown to immunosuppress fish and increase their susceptibility to diseases [192,193,194]. Interestingly, cortisol has been shown to be a strong inhibitor of NO production in goldfish macrophages [195]. Furthermore, treatment of a rainbow trout cell line with a combination of pro-inflammatory stimuli and cortisol results in heightened expression of interleukin-10, suggesting that the cortisol treatment overrides the induction of pro-inflammatory responses [8].

In mammals, IL-10 signals through IL10R1 and IL10R2, leading to activation of STAT3 and inhibition of pro-inflammatory cytokine expression and, thus, Th1 and M1 functions [196]. IL-10 is produced by virtually all leukocytes and is generated by macrophages in response to TLR engagement, GCs, and C-type lectin signaling [164]. An IL-10R1 has been identified in zebrafish, goldfish, and grass carp [197,198], as well as IL-10R2 in rainbow trout [199]. Notably, the goldfish IL-10 has been shown to down-regulate IFNγ stimulation of the ROI response and inflammatory gene expression of goldfish monocytes (Grayfer *et al.*, 2011b) [91], demonstrating an evolutionarily conserved regulatory role for this fish cytokine. Alternatively activated macrophage subtypes of mammals and teleosts are depicted in Figure 1.

## 6. Conclusions

Akin to their mammalian counterparts, macrophages of teleost fish exhibit a plethora of functional roles including those pertaining to homeostasis, as well as host immune defenses, and are largely governed by their respective tissue niches and microenvironments. Perturbations in these homeostatic environments by pathogens or injury polarize resident macrophage populations aptly towards the appropriate functionalities, with the sum of the cell types altering the physiology of the hosts towards an M1 inflammatory type, or the M2 resolution and repair types. Although these broad functional states have been demonstrated in fish macrophages, further research into the molecular regulation by both mammalian orthologs and novel fish-specific molecules will further the understanding of teleost macrophage functional regulation. Undoubtedly, the growing genetic resources of teleost animal models combined with transcriptomic/proteonomic/metabolomic technologies and live-imaging techniques of differentially activated teleost macrophages will be integral in deciphering the intricacies of macrophage functional regulation in teleosts.

## Abbreviations

Arg-1, arginase-1; AGM, aorta-gonad-mesonephros; CSF, colony stimulating factor; CSF-1R, colony stimulating factor-1 receptor; GM-CSF, granulocyte-macrophage colony-stimulating factor; EMP, erythromyeloid progenitors; GC, glucocorticoids; HSCs, hematopoietic stem cells; IDO, indoleamine 2,3-dioxygenase; IFN, interferon; IL, interleukin; M-CSF, macrophage-colony-stimulating factor; NK, natural killer cell; NO, nitric oxide; NRAMP1, natural resistance-associated macrophage protein 1; PAMPs, pathogen associated molecular patterns; PMA, phorbol myrystate acetate; PRR, pattern recognition receptor; rg, recombinant goldfish; RNI, reactive nitrogen intermediates; ROI, reactive oxygen intermediates; Slc11a1, Solute carrier 11 member 1; Stat, signal transducer of activation transcription factor; TGF, transforming growth factor; TLR, toll-like receptor; TNF, tumor necrosis factor.
